# Supplementary material for: The human visual system differentially represents subjectively and objectively invisible stimuli
Source: PLoS Biol. 2021 May 5;19(5):e3001241. doi: 10.1371/journal.pbio.3001241 (PMC8128378; doi:10.1371/journal.pbio.3001241)
Supplement: S1 Text — fMRI, functional magnetic resonance imaging; ROI, region of interest. (PDF) [file pbio.3001241.s001.pdf]

The human visual system differentially represents subjectively and objectively invisible stimuli

S1 Text

Timo Stein[1*], Daniel Kaiser[2], Johannes J. Fahrenfort[1,3], and Simon van Gaal[1]

[1] Brain and Cognition, Department of Psychology, University of Amsterdam, The Netherlands

[2] Department of Psychology, University of York, United Kingdom

[3] Cognitive Psychology, Department of Experimental and Applied Psychology, Vrije Universiteit Amsterdam, The Netherlands

***Correspondence**

Timo Stein, Brain and Cognition, Department of Psychology, University of Amsterdam, 1001 NK Amsterdam, The Netherlands.

Email: timo@timostein.de

Masking-efficiency experiment

S1 Fig shows normalized face-house discriminability and subjective visibility results from the masking-efficiency experiment. We fit a four-parametric logistic function to mean scores (*d'* and proportion "visible" presses, respectively) for every level of mask contrast that had been scaled between 0 and 1. Both measures exhibited a highly similar sigmoid dependence on mask contrast.

In the masking-efficiency experiment, at the three highest mask strengths, where average *d'* was below one, between-subject variability was low (SDs for mask contrast of 38, 61, and 100% were 0.41, 0.31, and 0.41, respectively, see **S1 Fig**): different observers performed in a narrow range for high mask contrast. We therefore calibrated mask contrast for the whole group of participants in the fMRI experiment based on the masking-efficiency experiment group data. An alternative approach is to calibrate mask contrast individually to allow for maximum signal strength for every participant. Such individual calibration requires reliable individual-subject estimates. However, the data from the masking-efficiency experiment revealed low reliability for individual differences at high mask contrasts. **S1 Table** shows correlations of face-house discrimination performance (in proportion correct, to avoid extreme *d'* values for small number of trials) between the nine different mask contrasts. Note that for the three mask levels where *d'* was below one correlations are below $r = .17$. The diagonal of **S1 Table** shows within-condition reliability estimates. These were calculated by correlating performance from two randomly determined halves of the data set. This process was repeated 100 times; the resulting correlation coefficients were averaged (after Fisher z-transformation), and the mean score was back-transformed. While reliability was generally high when overall performance was above a *d'* of one, for the three highest masking strength reliability was low (below $r = .19$). With such low reliability for individual differences participant-based adjustment of mask contrast (for example, via an adaptive staircase procedure) would capitalize on noise. This, in turn, would have risked above-chance performance for some participants in the fMRI experiment. To avoid issues with post-hoc data selection[1], such as regression to the mean[2], we considered group-based calibration of mask contrast superior for the purpose of our study. Note that these considerations are naturally limited to the particular stimuli and sandwich masking paradigm adopted in our study; with other presentation methods, such as continuous flash suppression[3], individual stimulus calibration may be preferable.

Evidence for objective invisibility

For the interpretation of our fMRI results in our *obj-inv* condition it is important to test whether faces and houses were truly indiscriminable (objectively invisible). In the main manuscript, we report that discrimination performance ($M = 0.02$, $SD = 0.24$) did not differ significantly from chance, with moderate evidence for the null hypothesis of chance-level discrimination ($t_{(42)} = 0.45$, $p = .33$ (one-tailed), $d_z = 0.07$, $BF_{0+} = 4.13$).

Standard statistical significance testing. Another approach that has been adopted in the literature on unconscious processing is to test every participant's performance against chance level. **S2 Fig** shows face-house discrimination as proportion correct. One-tailed binomial tests comparing every participant's accuracy against the chance level of 0.5 revealed (only) one participant whose performance was significantly above the chance level (p = .028). When carrying out 43 tests, some are expected to return a significant result. In fact, the distribution of p-values from the 43 binomial tests (**S1 Fig**) looks just as one would expect if values were randomly sampled from a binomial distribution with a probability of 0.5 success for each trial. However, as discussed in the main paper, although absence of awareness is commonly demonstrated by showing that performance did not exceed what is expected by chance (p > .05), a non-significant result simply means that the observed effect is not very surprising under the null hypothesis of no effect, but cannot be taken as support for the null hypothesis.

Furthermore, p-values increase with decreasing power and increasing variance in the awareness measure, such that a non-significant result is not particularly surprising if the awareness measure is collected in few trials and small participant samples (i.e. with low power)[4,5]. To address these issues, we tested a much larger sample than previous fMRI studies, measured awareness in every trial during scanning (average of 196 trials per participant to calculate *d'* in the *obj-inv* condition), and did not exclude any participants based on their awareness data (which can severely inflate estimates of unconscious processing due to regression to the mean[2]). Although our fMRI study adopted the highest criteria for demonstrating unconscious processing of objectively invisible stimuli to date, approaches other than the standard frequentist significance-testing approach are required to provide evidence for absence of awareness. Two such alternative approaches are *Bayesian statistics* (as reported in the main paper) and *equivalence tests*.

Equivalence tests. Equivalence tests based on the two one-sided tests (TOST) procedure can be used to determine if an observed effect is surprisingly small under the assumption that a true effect, at least as large as a specific smallest effect size of interest (SESOI), exists[6]. In the context of unconscious processing, setting an SESOI represents a non-trivial, subjective call, as it requires determining the smallest *d'* score that can still reasonably be considered objectively invisible. Setting the SESOI (arbitrarily) to a *d'* score of 0.10 the TOST procedure (using the spreadsheet TOST calculators provided by Lakens and colleagues[6]) revealed that the observed effect was significant within the equivalent bounds of –0.1 and 0.1 scale points, $t_{(42)} = -2.23$, $p = .016$. We note that when setting the SESOI to a *d'* score of 0.15, the TOST would return $p < .001$; when setting the SESOI to a *d'* score of 0.05, however, the TOST would return $p = .19$. These results provide more insight about the uncertainty in our *obj-inv* data. While we cannot reasonably claim that discrimination performance was surprisingly low if the true effect was *d'* = 0.05, our data are unlikely if the true effect was *d'* = 0.10 or larger.

Bayesian statistics. The Bayesian statistical framework allows estimating how likely the observed data are under the null hypothesis, compared to the alternative hypothesis, and has thus particular utility in the study of unconscious processes[7]. While the null hypothesis in this framework is a point estimate (zero), Bayesian *t*-tests also require a non-trivial, subjective call in specifying the prior distribution for the alternative hypothesis. Estimates of the probability of the null hypothesis relative to the alternative hypothesis (BF_{0+}) depend on this prior. In the main paper, we used the default prior provided in the JASP software package (Cauchy distribution centered on zero and scale 0.707), and this resulted in "moderate" evidence for the null hypothesis ($BF_{0+} = 4.13$). A wider prior (assigning more weight to larger effect sizes) would result in higher probability of the null hypothesis (e.g. with a Cauchy distribution with a scale of one, $BF_{0+} = 5.67$). Conversely, a narrower prior (assigning more weight to smaller effect sizes) would results in lower probability of the null hypothesis (e.g. with a normal distribution with $M = 0$ and $SD = 0.5$, $BF_{0+} = 2.34$). Rather than allowing definite conclusions about absence of awareness, our results highlight the statistical uncertainty inherent in demonstrations of unconscious processing, and invite discussions about reasonable prior assumptions and about which (bands of) effect sizes represent conclusive evidence for presence vs. absence of conscious awareness.

Univariate ROI results

We also conducted standard univariate ROI analyses (same ROI definitions as for the MVPA analyses). Here, for every participant and ROI we used the localizer data to determine the 100 most face-responsive voxels and the 100 most house-selective voxels. We then calculated for every visibility condition in the main experiment the mean difference in activity evoked by faces vs. houses in the face-responsive voxels and the mean difference in activity evoked by houses vs. faces in the house-responsive voxels. These differences were then averaged to yield a mean beta difference ($\Delta\beta$) reflecting how well a region's activity in the main experiment distinguished its preferred category from the non-preferred category; $\Delta\beta$ was used as a univariate measure of category information. Univariate results were similar to the MVPA results reported in the main paper (compare **S3 Fig** with **Fig 2**).

Category information in visual cortex. As for the MVPA analyses, there were marked differences between the regions, with overall greater response differences in LOC than in V1 ($F_{(1, 42)} = 156.82$, $p < .001$, $\eta_p^2 = .79$, $BF_{10} = 1.35\times10^{22}$), as well as response differences between visibility conditions $F_{(3, 126)} = 56.44$, $p < .001$, $\eta_p^2 = .57$, $BF_{10} = 2.69\times10^{27}$), and these differences were more pronounced in LOC than in V1 (interaction, $F_{(3, 126)} = 66.72$, $p < .001$, $\eta_p^2 = .61$, $BF_{10} = 2.74\times10^{18}$). Separate one-tailed *t*-tests for every region and every visibility condition revealed significant category information in V1 for *obj-vis* ($t_{(42)} = 3.57$, $p < .001$, $d_z = 0.55$, $BF_{+0} = 65.68$), *subj-vis* ($t_{(42)} = 3.41$, $p < .001$, $d_z = 0.52$, $BF_{+0} = 43.36$), and *obj-inv* ($t(_{42}) = 2.04$, $p = .024$, $d_z = 0.31$, $BF_{+0} = 2.09$), but not for *subj-inv* ($t_{(42)} = 0.36$, $p = .36$, $d_z = 0.06$, $BF_{0+} = 4.48$). LOC shoed significant category information in all visibility conditions, *obj-vis* ($t_{(42)} =$

22.77, $p < .001$, $d_z = 3.47$, $BF_{+0} = 2.18×10^{22}$), *subj-vis* ($t_{(42)} = 12.11$, $p < .001$, $d_z = 1.85$, $BF_{+0} = 4.79×10^{12}$), *subj-inv* ($t_{(42)} = 4.86$, $p < .001$, $d_z = 0.74$, $BF_{+0} = 2.43×10^{3}$), and also in *obj-inv* ($t_{(42)} = 3.17$, $p = .001$, $d_z = 0.48$, $BF_{+0} = 23.74$).

Similarly, for category-selective regions, category information was more pronounced in anterior (FFA/PPA) than in posterior (OFA/OPA) areas, ($F_{(1, 42)} = 111.32$, $p < .001$, $\eta_p^2 = .73$, $BF_{10} = 1.23×10^{18}$); there were also significant differences between visibility conditions ($F_{(3, 126)} = 92.41$, $p < .001$, $\eta_p^2 = .69$, $BF_{10} = 5.18×10^{46}$), and a significant interaction ($F_{(3, 126)} = 41.51$, $p < .001$, $\eta_p^2 = .50$, $BF_{10} = 4.25×10^{9}$). In OFA/OPA category information was significant in all conditions, *obj-vis* ($t_{(42)} = 11.98$, $p < .001$, $d_z = 1.82$, $BF_{+0} = 3.47×10^{12}$), *subj-vis* ($t(42) = 7.47$, $p < .001$, $d_z = 1.14$, $BF_{+0} = 7.61×10^{6}$), *subj-inv* ($t_{(42)} = 4.30$, $p < .001$, $d_z = 0.66$, $BF_{+0} = 478.01$), and *obj-inv* ($t_{(42)} = 2.96$, $p = .003$, $d_z = 0.45$, $BF_{+0} = 14.37$). Also in FFA/PPA category information was significant in all visibility conditions, but as for the MVPA results, strong evidence for category information was obtained only for *obj-vis* ($t_{(42)} = 15.92$, $p < .001$, $d_z = 2.43$, $BF_{+0} = 4.56×10^{16}$), *subj-vis* ($t_{(42)} = 13.22$, $p < .001$, $d_z = 2.02$, $BF_{+0} = 8.10×10^{13}$), and *subj-inv* ($t_{(42)} = 6.04$, $p < .001$, $d_z = 0.92$, $BF_{+0} = 8.95×10^{4}$), but not for *obj-inv* ($t_{(42)} = 1.76$, $p = .043$, $d_z = 0.27$, $BF_{+0} = 1.29$).

Differences between visibility conditions. In both early visual cortex (V1) and object-selective visual cortex (LOC) subjective visibility was associated with greater category information than subjective invisibility (V1: $t_{(42)} = 2.46$, $p = .018$, $d_z = 0.38$, but $BF_{10} = 2.39$; LOC: $t_{(42)} = 8.44$, $p < .001$, $d_z = 1.29$, $BF_{10} = 7.57×10^{7}$), but this difference was more pronounced in LOC (interaction, $F_{(1, 42)} = 34.79$, $p < .001$, $\eta_p^2 = .45$, $BF_{10} = 1.22×10^{3}$). Also the way of establishing invisibility influenced the two areas differently (interaction, $F_{(1, 42)} = 9.83$, $p = .003$, $\eta_p^2 = .19$, $BF_{10} = 5.59$). While in V1 there was no significant difference in category information for subjectively and objectively invisible stimuli ($t_{(42)} = -1.26$, $p = .21$, $d_z = 0.19$, $BF_{01} = 2.89$), in LOC category information was significantly greater for subjectively than for objectively invisible stimuli ($t_{(42)} = 2.27$, $p = .029$, $d_z = 0.35$, but $BF_{10} = 1.64$).

In both posterior and anterior category-selective regions subjective visibility was associated with greater category information than subjective invisibility (OFA/OPA: $t_{(42)} = 4.24$, $p < .001$, $d_z = 0.64$, $BF_{10} = 1.93×10^{3}$; FFA/PPA: $t_{(42)} = 6.42$, $p < .001$, $d_z = 0.98$, $BF_{10} = 2.73×10^{7}$), this difference was more pronounced in FFA/PPA (interaction, $F_{(1, 42)} = 21.05$, $p < .001$, $\eta_p^2 = .33$, $BF_{10} = 14.37$). Also the method of establishing invisibility had a different effect on the two ROIs (interaction, $F_{(1, 42)} = 23.01$, $p < .001$, $\eta_p^2 = .35$, $BF_{10} = 62.34$). While there was no significant difference between subjectively and objectively invisible stimuli in OFA/OPA ($t_{(42)} = 0.79$, $p = .437$, $d_z = 0.12$, $BF_{01} = 2.31$), FFA/PPA showed greater category information for subjectively than for objectively invisible stimuli ($t_{(42)} = 4.23$, $p < .001$, $d_z = 0.64$, $BF_{10} = 597.15$).

Posterior-anterior category preference gradient. The univariate ROI data also showed a posterior-anterior gradient. For *obj-vis*, *subj-vis*, and *subj-inv* category information increased from V1 to LOC (all $t_{(42)} > 4.50$, $p < .001$, $d_z > 0.59$, $BF_{10} > 75.25$, **S3 Fig**), and from OFA/OPA to FFA/PPA (all $t_{(42)}$

> 5.11, $p < .001$, $d_z > 0.77$, $BF_{10} = 2.62 \times 10^3$, **S3 Fig**). For *obj-inv*, this gradient was virtually absent; category information did not differ significantly between V1 and LOC ($t_{(42)} = 0.06$, $p = .95$, $d_z < 0.01$, $BF_{01} = 6.05$) or between OFA/OPA and FFA/PPA ($t_{(42)} = 0.07$, $p = .95$, $d_z = 0.01$, $BF_{01} = 6.04$).

Different ROI definitions

For the univariate analyses and for the ROI analyses reported in the main paper we selected the 100 most face-responsive and the 100 most house-responsive voxels for each ROI, as determined by the localizer scan. To ensure that our results did not depend on this particular ROI size of 200 voxels, we repeated the MVPA correlation analyses for other ROI definitions (including between 10 [i.e. the 5 most face-responsive, and the 5 most house-responsive voxels in the localizer] and 500 voxels, in steps of 10). As can be seen in **S4 Fig**, in all ROIs except for V1, overall pattern discrimination (category information Δr) dramatically increased with smaller ROI size. This was especially the case for the *obj-vis* and *subj-vis* conditions, and in LOC and in FFA/PPA also for *subj-inv.* ROI size had little influence on category information for the *obj-inv* condition. Most importantly, the patterns of differences between visibility conditions as reported in the main paper were robust across ROI definitions.

Main-experiment searchlights

All analyses reported in the main paper relate brain activity in the main experiment to the independent localizer scan, where participants did a simple one-back task on unmasked faces and houses presented for 750 ms each in a block design. We designed the study for this particular analysis strategy, because it is statistically powerful and isolates perceptual representations from later cognitive processes related to the task, decision, and motor processes[8,9]. Analyses based on the data from the main experiment alone cannot exclude such influences, for example, in these analyses button presses are confounded with the different stimulus and visibility conditions.

Category information. Accordingly, additional searchlight analyses based on the main-experiment data alone revealed category information in motor cortex, driven by the responses given to the presented categories (**S5 Fig**). For these searchlights, the main experiment data were split into two sets (all possible splits of two and three runs). For every visibility conditions we repeatedly calculated within- and between-category pattern correlation differences for a moving sphere with a radius of five voxels (524 voxels in the sphere) which was centered on every voxel in the functional images of every participant. Correlations from all possible splits were then averaged to yield the searchlight maps.

These analyses revealed areas carrying significant category information across the whole brain (corrected for multiple comparisons via false discovery estimation, $p < .05$) in the *obj-vis*, *subj-vis*, and *subj-inv* condition, but not in the *obj-inv* condition (**S5 Fig**). In all three visibility conditions clusters with significant category information were located in bilateral fusiform gyrus, but most prominently in

bilateral motor cortex (highlighted in the slices shown in **S5 Fig**). Such motor cortex pattern discrimination reflects the correlation between stimulus category and participant's button presses for those conditions in which button presses (perceptual discrimination) carried information about stimulus category (when discrimination performance is above chance). Accordingly, additional frontal cortex clusters (in right inferior frontal gyrus, orbital parts of right superior and left middle frontal gyrus) revealed in the contrast between *subj-inv* and *subj-vis* (**S5 Fig**) are difficult to interpret. Remarkably, there was no evidence for category information in motor cortex in the *obj-inv* condition, indicating that in this condition (neural representations of) button presses were indeed unrelated to stimulus category, thus further supporting the notion that stimuli were fully indiscriminable.

Visibility irrespective of category. In addition to occipitotemporal areas, several previous fMRI studies reported correlates of conscious visual perception in a frontoparietal network[10,11]. These studies compared brain activity evoked by visible and invisible conditions, independent of stimulus category or content, assessing visibility either with objective measures (e.g. by comparing weakly vs. strongly masked stimuli[12]) or with subjective measures of visibility (e.g. by comparing similar or identical stimuli judged as "visible" vs. "invisible"[11]). To test for such frontoparietal involvement in content-independent conscious perception ("conscious access" or "report"), we conducted searchlight analyses that revealed brain areas where activity distinguished between visibility conditions, independent of object category. For these searchlights, we again split the main experiment data into two sets (all possible splits of two and three runs). Separately for the objective and for the subjective condition we repeatedly calculated within-visibility (visible/visible and invisible/invisible) and between-visibility (visible/invisible) pattern correlations for a moving sphere with a radius of five voxels (524 voxels in the sphere) which was centered on every voxel in the functional images of every participant. Correlations from all possible splits were then averaged to yield the searchlight maps.

In both the objective and subjective condition activity in many brain areas distinguished between visible and invisible trials (corrected for multiple comparisons via false discovery estimation, $p < .05$), including early visual cortex, fusiform and inferior temporal gyrus, parietal cortex (e.g. inferior parietal lobule, precuneus, angular gyrus, postcentral gyrus), frontal cortex (e.g. motor cortex, inferior frontal gyrus, superior frontal gyrus, middle frontal gyrus, anterior cingulate), and anterior cingulate cortex. The sagittal views of the right hemisphere in **S6 Fig** highlight significant pattern correlations in ventrotemporal areas, parietal cortex, and inferior frontal gyrus in both objective and subjective conditions. These results could be considered evidence for frontoparietal involvement in (content-independent) conscious perception. Note, however, that in these analyses based on the main-experiment data, post-perceptual processes (related to the task, decision, and motor processes) and stimulus strength (mask contrast) are confounded with differences in visibility

References

1. Schmidt, T. Invisible stimuli, implicit thresholds: Why invisibility judgments cannot be interpreted in isolation. *Adv. Cogn. Psychol.* **11**, 31–41 (2015).
2. Shanks, D. R. Regressive research: The pitfalls of post hoc data selection in the study of unconscious mental processes. *Psychon. Bull. Rev.* **24**, 752–775 (2017).
3. Hesselmann, G., Darcy, N., Ludwig, K. & Sterzer, P. Priming in a shape task but not in a category task under continuous flash suppression. *J. Vis.* **16**, 1–17 (2016).
4. Vadillo, M. A., Konstantinidis, E. & Shanks, D. R. Underpowered samples, false negatives, and unconscious learning. *Psychon. Bull. Rev.* **23**, 87–102 (2016).
5. Vadillo, M. A., Linssen, D., Orgaz, C., Parsons, S. & Shanks, D. R. Unconscious or underpowered? Probabilistic cuing of visual attention. *J. Exp. Psychol. Gen.* **149**, 160–181 (2020).
6. Lakens, D., Scheel, A. M. & Isager, P. M. Equivalence testing for psychological research: A tutorial. *Adv. Methods Pract. Psychol. Sci.* **1**, 259–269 (2018).
7. Dienes, Z. How Bayesian statistics are needed to determine whether mental states are unconscious. in *Behavioral Methods in Consciousness Research* (ed. Overgaard, M.) 199–220 (Oxford Academic, 2015).
8. Peelen, M. V., Fei-Fei, L. & Kastner, S. Neural mechanisms of rapid natural scene categorization in human visual cortex. *Nature* **460**, 94–97 (2009).
9. Peelen, M. V. & Kastner, S. A neural basis for real-world visual search in human occipitotemporal cortex. *Proc. Natl. Acad. Sci. U. S. A.* **108**, 12125–12130 (2011).
10. Dehaene, S., Changeux, J. P., Naccache, L., Sackur, J. & Sergent, C. Conscious, preconscious, and subliminal processing: A testable taxonomy. *Trends Cogn. Sci.* **10**, 204–211 (2006).
11. Rees, G. Neural correlates of the contents of visual awareness in humans. *Philos. Trans. R. Soc. B Biol. Sci.* **362**, 877–886 (2007).
12. Dehaene, S. *et al.* Cerebral mechanisms of word masking and unconscious repetition priming. *Nat. Neurosci.* **4**, 752–758 (2001).
