# Supplementary material for: The human visual system differentially represents subjectively and objectively invisible stimuli
Source: PLoS Biol. 2021 May 5;19(5):e3001241. doi: 10.1371/journal.pbio.3001241 (PMC8128378; doi:10.1371/journal.pbio.3001241)
Supplement: S1 Table — The diagonal (in bold) shows within-condition reliability estimates, which were calculated by repeatedly correlating performance from 2 randomly determined halves of the data set. The other cells show correlations between performance from different mask conditions. (PDF) [file pbio.3001241.s002.pdf]

Table S1

Reliability estimates of face-house discrimination performance in the masking-efficiency experiment

Mask contrast	2%	3%	5%	9%	14%	23%	38%	61%	100%
2%	**.48**								
3%	.81	**.78**							
5%	.55	.69	**.37**						
9%	.67	.69	.82	**.68**					
14%	.62	.73	.64	.66	**.75**				
23%	.28	.33	.29	.37	.52	**.71**			
38%	.15	.28	.45	.45	.37	.78	**.01**		
61%	−.17	−.21	.11	.20	−.06	−.12	−.07	**−.25**	
100%	.42	.28	.08	.30	.22	−.07	−.01	.16	**.18**

Note. The diagonal (in bold) shows within-condition reliability estimates, which were calculated by repeatedly correlating performance from two randomly determined halves of the data set. The other cells show correlations between performance from different mask conditions.
