# Supplementary figures and images for: The human visual system differentially represents subjectively and objectively invisible stimuli
Source: PLoS Biol. 2021 May 5;19(5):e3001241. doi: 10.1371/journal.pbio.3001241 (PMC8128378; doi:10.1371/journal.pbio.3001241)

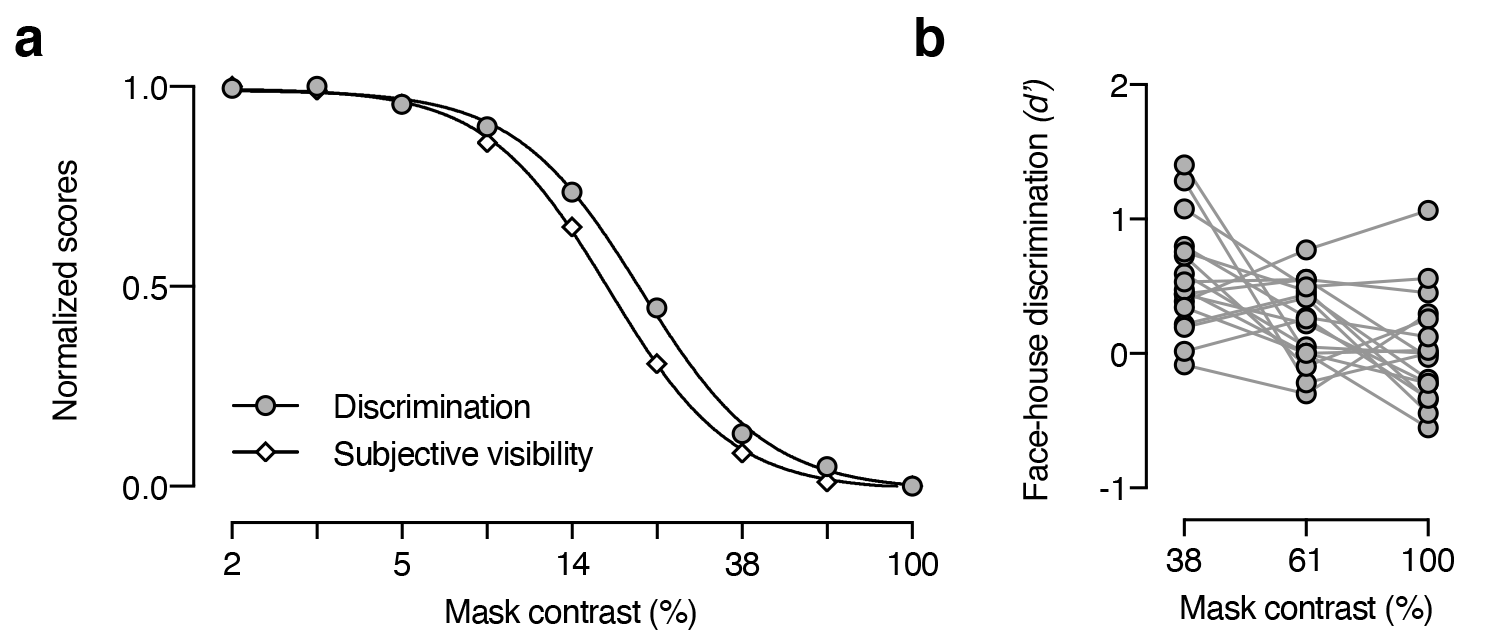

Supplement: S1 Fig — (a) Normalized results. For every level of mask contrast mean face-house discriminability and mean subjective visibility were scaled between 0 and 1, and a logistic function was fit to the resulting normalized scores. (b) Face-house discriminability for individual participants at the three highest mask contrasts (where mean d’ was below 1) in the masking efficiency experiment. Data underlying this figure are available on OSF (https://osf.io/qus5v/). (TIF) [file pbio.3001241.s003.tif]

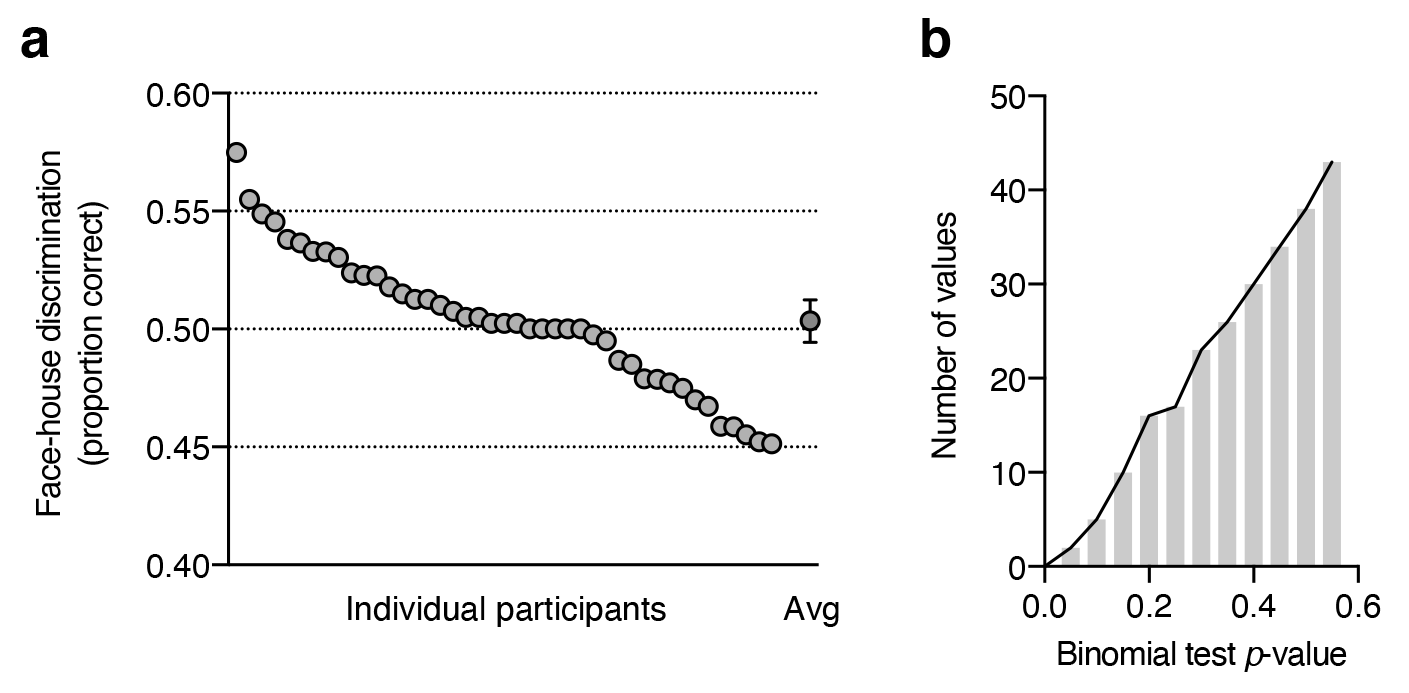

Supplement: S2 Fig — (a) Individual participant’s face-house discrimination performance in the obj-inv condition as proportion correct. Every gray circle represents a participant, and the red circle shows the group mean with its 95% confidence interval. (b) Histogram showing the cumulative distribution of p-values from the 1-sided binomial tests of face-house discrimination accuracy in the obj-inv condition. Data underlying this figure are available on OSF (https://osf.io/qus5v/). fMRI, functional magnetic resonance imaging; obj-inv, objectively invisible. (TIF) [file pbio.3001241.s004.tif]

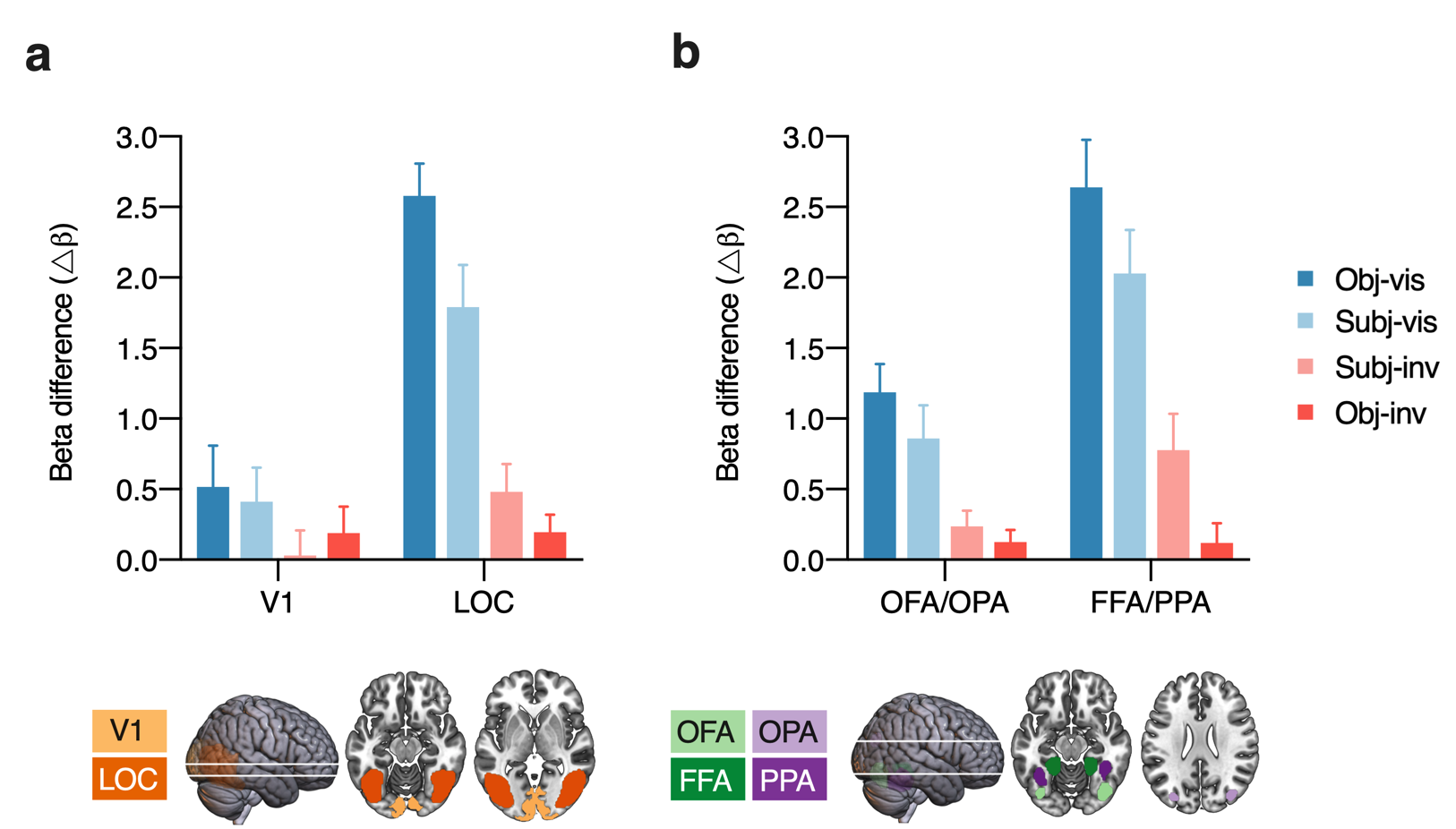

Supplement: S3 Fig — Responses to a voxel’s preferred category vs. its non-preferred category (as determined by the independent localizer) for the four visibility conditions in the main experiment, averaged across the voxels in each brain region. Bars show the mean beta difference between preferred and non-preferred categories (Δβ). Error bars represent 95% confidence intervals. (a) Results for early visual cortex (V1) vs. object-selective visual cortex (LOC). (b) Results for posterior category-selective areas (OFA/OPA) vs. anterior category-selective areas (FFA/PPA). Data underlying this figure are available on OSF (https://osf.io/qus5v/). FFA, fusiform face area; fMRI, functional magnetic resonance imaging; LOC, lateral occipital complex; OFA, occipital face area; OPA, occipital place area; PPA, parahippocampal place area; V1, primary visual cortex. (TIFF) [file pbio.3001241.s005.tiff]

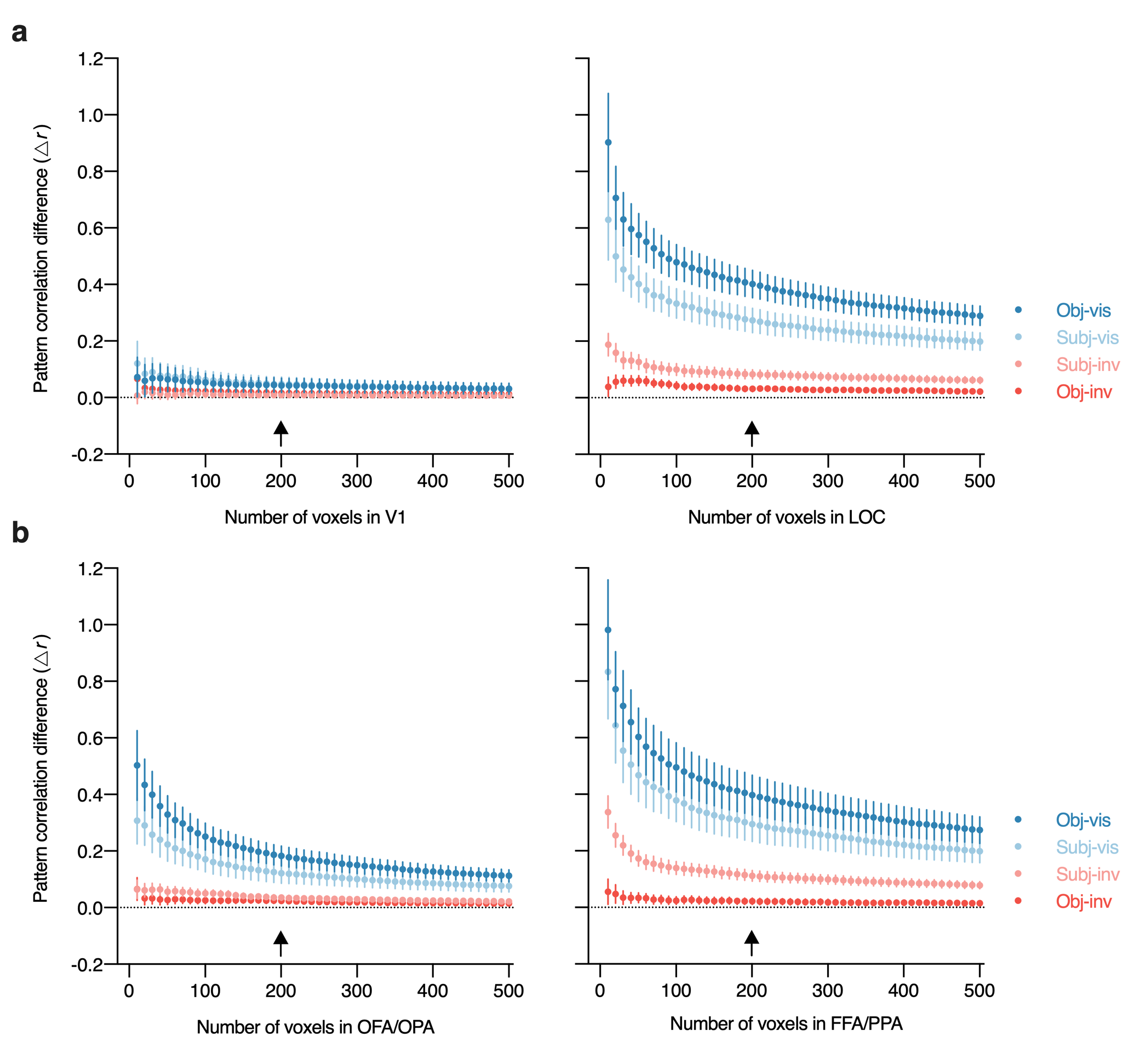

Supplement: S4 Fig — Multivoxel pattern correlations between the four visibility conditions in the main experiment and the independent localizer, for a range of different ROI definitions (containing the 10–500 most face- and house-responsive voxels). Symbols show the mean difference between within-category and between-category multivoxel pattern correlations (Δr). For better readability, error bars represent SEMs. Arrows indicate the ROI definition adopted for the results presented in the main paper. (a) Results for early visual cortex (V1, left panel) vs. object-selective visual cortex (LOC, right panel). (b) Results for posterior category-selective areas (OFA/OPA, left panel) vs. anterior category-selective areas (FFA/PPA, right panel). Data underlying this figure are available on OSF (https://osf.io/qus5v/). FFA, fusiform face area; LOC, lateral occipital complex; OFA, occipital face area; OPA, occipital place area; PPA, parahippocampal place area; ROI, region of interest; V1, primary visual cortex. (TIFF) [file pbio.3001241.s006.tiff]

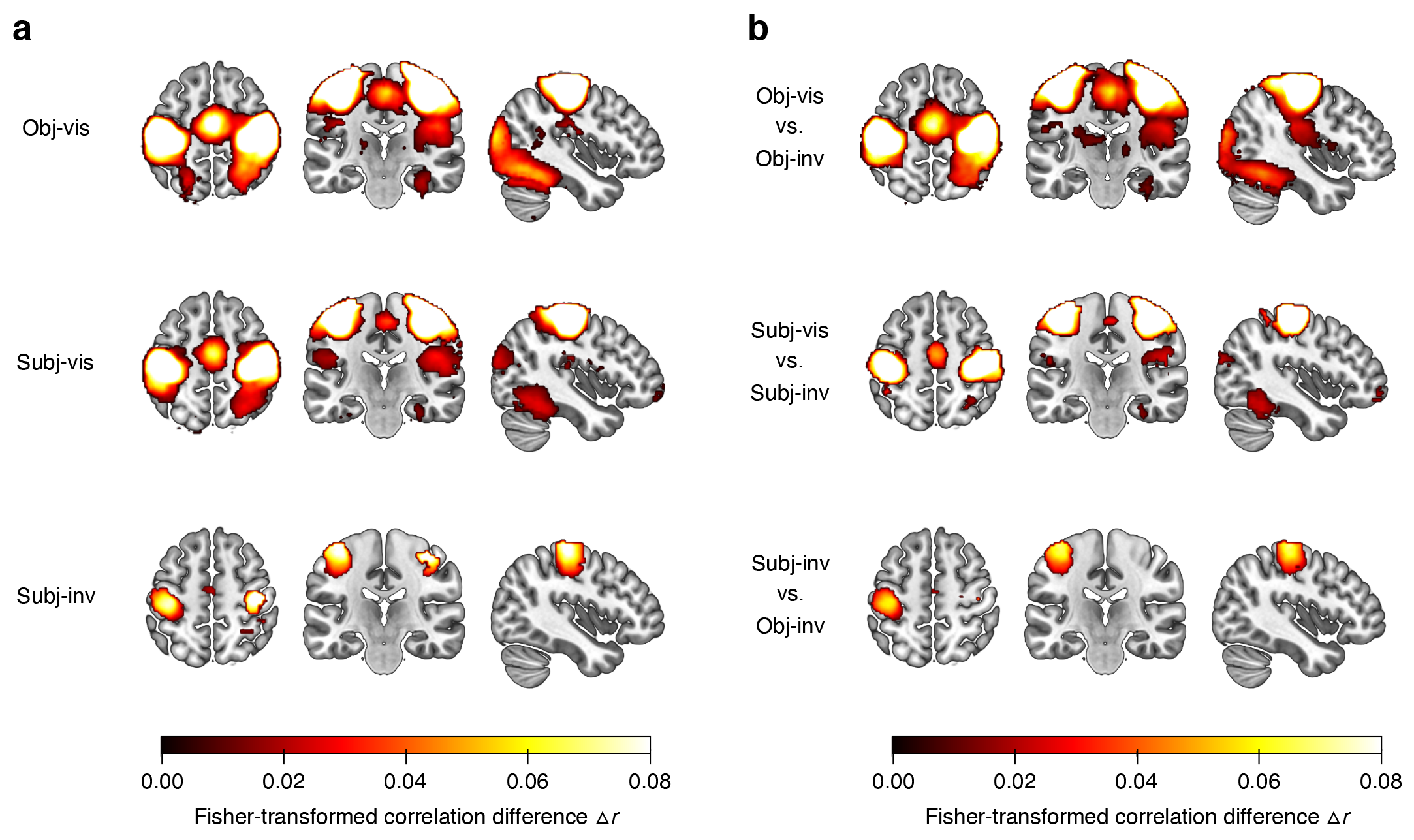

Supplement: S5 Fig — (a) Results from the additional searchlight analyses of the main experiment only, showing clusters with significant category information across the whole brain, separately for the different visibility conditions. Slices were selected to highlight motor cortex. Only voxels surviving multiple comparison correction via false discovery estimation (p < 0.05) are shown. For the objectively invisible condition, no clusters survived this statistical threshold. (b) Searchlight results showing the effect of subjective visibility by comparing the subj-vis to the subj-inv condition and the effect of method for establishing invisibility by comparing the subj-inv to the obj-inv condition. Data underlying this figure are available on OSF (https://osf.io/qus5v/). obj-inv, objectively invisible; subj-inv, subjectively invisible; subj-vis, subjectively visible. (TIF) [file pbio.3001241.s007.tif]

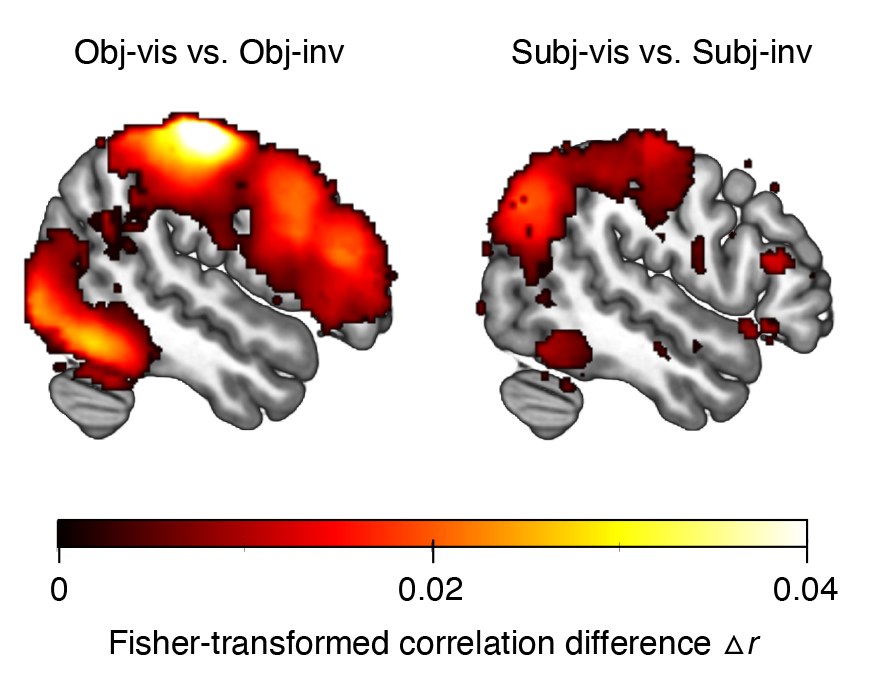

Supplement: S6 Fig — Results from searchlight analyses of the main experiment only, showing clusters with significant information about stimulus visibility across the whole brain (independent of stimulus category), separately for the objective condition (comparing obj-vis to obj-inv) and for the subjective condition (comparing subj-vis to subj-inv). Slices show the right hemisphere and were selected to highlight ventrotemporal regions, parietal cortex, and inferior frontal gyrus in both conditions. Only voxels surviving multiple comparison correction via false discovery estimation (p < 0.05) are shown. Data underlying this figure are available on OSF (https://osf.io/qus5v/). obj-inv, objectively invisible; obj-vis, objectively visible; subj-inv, subjectively invisible; subj-vis, subjectively visible. (TIF) [file pbio.3001241.s008.tif]
