# Supplementary material for: The human visual system differentially represents subjectively and objectively invisible stimuli
Source: PLoS Biol. 2021 May 5;19(5):e3001241. doi: 10.1371/journal.pbio.3001241 (PMC8128378; doi:10.1371/journal.pbio.3001241)
Supplement: Attachment — Submitted filename: rebuttal.docx [file pbio.3001241.s009.docx]

**Academic editor**

Abstract:

"We show that neural representations of objectively invisible faces and houses

are limited to visual (shape-related) object properties, while subjectively invisible stimuli

are processed up to more abstract, categorical levels of representation"- I think the contrast between visual and abstract is misleading- it implies that category-selective areas are not visual- which is unfounded. The authors should use more neutral terms- such as early or lower-level vs. higher level visual areas.

**Response:** We changed the wording accordingly, both in the Abstract and in the first paragraph of the Discussion (“early, lower-level visual areas”, “higher-level visual cortex”, “object- and category selective visual areas”).

Results:

Figure 1:

It will be informative if the authors show the fit between panels b and c- by normalizing the values between 0 and 100% and superimposing the two curves. The fit should be pointed out, as well as the highly non-linear (sigmoid) nature of the dependence on mask contrast.

**Response:** We agree that the fit between the objective and subjective curves is striking and we included a figure with overlaid, normalized values in the Supplement, and we highlight the non-linear sigmoid dependence on mask contrast for both measures. For the main manuscript, we found it important to clearly show the actual (non-normalized) values (d’ and visibility proportions), and thus kept the two separate graphs/axes.

Discussion:

General points:

It is important that the authors discuss the meaning of the "stronger representation" to use their terminology when analyzed through multivariate correlation patterns. Specifically- a stronger vs weaker representation may result from at least two main sources: first, a weaker univariate activation- i.e. lower signal to noise responses in each voxel. Second- it may result from a change in the pattern of activation elicited during the weak vs strong responses-resulting in weaker and stronger similarity to the bench mark patterns. This point needs to be clarified- and kept in mind when interpreting the results. Just as an example is the following conclusion: "The present findings will resonate with the idea that subjectively invisible stimuli are processed in a way that is qualitatively similar to (clearly) visible stimuli"- In this statement the authors, presumably, assume that the patterns of activation for the subjectively invisible and visible stimuli are similar-differing only in signal amplitude. However this conclusion ignores the fact that the massive reduction in representation strength for subjectively invisible stimuli may be the result of a corresponding change in the pattern of activation making it dissimilar to the benchmark pattern.

**Response:** In our revision, we highlight these two possible mechanisms underlying differences in multivariate correlation patterns at the beginning of the Results section (“Note that differences in multi-voxel pattern correlations can result from changes in the fine-grained activation patterns or from differences in univariate activation [for complimentary univariate analyses see Supplementary material and Figure S3].)”. Furthermore, in the Discussion we make clear that our findings could reflect differences in patterns without a concurrent difference in response amplitude. However, as we obtained almost equivalent results in our univariate ROI analyses (reported in the Supplement) we consider differences in response amplitude the more likely explanation for differences in multivariate correlation patterns (“Although differences in multi-voxel pattern correlations could reflect differences in response amplitude or changes in activation patterns, we obtained almost identical results in an univariate ROI analysis [see Supplementary Material and Figure S3], and thus consider differences in response amplitude the more likely account for differences between visibility conditions.”).

A second major methodological issue that should be discussed is the critical difference in the response properties to visible stimuli in early vs high order cortex- specifically in this case to the mask vs. target stimuli. It is quite likely that the weak category representations they find in V1 during the visible conditions is due to literally masking the category information by the much stronger mask response- since the fMRI method is too sluggish to temporally separate out the target response from the far larger response to the masks. This can easily lead to an erroneous underestimation of the low-level differences between the categories in V1 even for visible stimuli. This point should be addressed by the authors.

**Response:** We agree that the comparably weak category representations in V1 even during the visible conditions are remarkable. However, we believe this is unlikely to be caused by responses to the masks. On this account, there should be a strong effect of mask contrast on category representations in V1. However, while mask contrast strongly modulated category representations in higher-level visual cortex, there was no such effect in V1. For example, V1 category information did not differ between obj-vis (2% mask contrast) and subj-vis (8% mask contrast). Similarly, there was no difference in V1 category information between subj-inv (12% mask contrast) and obj-inv (100% mask contrast). With 2% contrast in the obj-vis condition masks were hardly perceivable (e.g. see Figure 1b). Accounts based on masking strength thus provide a poor fit to the V1 data. Rather, reduced category information in V1 reflects the region’s sensitivity to low-level stimulus properties, which were equated in our face and house stimuli.

Specific points:

Before jumping into the discussion of the brain activations associated with the invisible conditions- it is important that the authors will first note the main, and very striking, effects demonstrated by their study. In particular-the very dramatic change in the "visibility effect"- i.e. the difference between visible vs invisible conditions- as one moves from early (essentially no difference) to high order visual cortex (massive difference). Similarly they should point in this context the abolishment of such visibility effect in frontal cortex.

**Response:** We changed the first paragraph of our Discussion section to follow the suggested structure, starting with the second sentence: “Using fMRI we found that both objective and subjective visibility were associated with a clear posterior-to-anterior gradient: While there was little effect of visibility on category information in early visual cortex, higher-level visual cortex was strongly modulated by visibility. In frontal cortex we did not obtain evidence for such effects of visibility on category representations.”

"Rendering stimuli perceptually indiscriminable requires very low stimulus strength" This is incorrect- there are cases where strong stimuli can be rendered invisible - such as binocular rivalry, crowding and motion-induced blindness.

**Response:** Thanks for spotting this. We agree that some presentation methods allow for higher input strength (at the retina) than backward masking. In our revised manuscript, we changed the sentence to refer specifically to visual masking techniques (“Rendering stimuli perceptually indiscriminable through visual masking requires very low stimulus strength”).

"above-chance performance for subjectively invisible stimuli simply means that

these stimuli were in fact not invisible"-

A possible analysis that may help clarify this conundrum could be to analyze separately those trials in which participants marked the target as invisible but guessed the category correctly vs. those subjectively invisible trials in which the participants guessed incorrectly.

**Response:** Although we appreciate the suggestion, we did not have enough data points for this analysis. When splitting the subjectively invisible condition according to accuracy and category (as required for this analysis), the median (mean) number of trials per cell ranged between 12 (17) and 84 (84), with 11 subjects having only ≤5 trials in one of the cells, and 18 subjects having ≤ 10 trials in one of the cells (implying that not all runs could have been modeled). Determining whether blindsight-like above-chance performance for subjectively invisible stimuli reflects unconscious perception or a conservative response criterion in the subjective measure has been notoriously difficult. One promising avenue for future studies that we are now highlighting in the Discussion (new paragraph discussing subjective measures) is to adopt recently developed criterion-free measures of subjective awareness such as meta-*d’* based on confidence ratings. We have added an extra paragraph to the discussion section, also in response to comments of the other two reviewers, discussing this issue:

"objectively invisible stimuli can be decoded from patterns of fMRI activity in human visual cortex- to be fair to their own data the authors should add "albeit at a drastically lower strength than visible stimuli"

**Response:** Thank you. We added the proposed qualification to the sentence.

**Reviewer #2**

1. On page 14 (middle of first full paragraph), the authors state, "Category information could be decoded from early visual cortex, from object-selective LOC, from the (largely overlapping) category-selective areas in lateral occipital cortex (OFA/OPA), and, less robustly, from ventrotemporal areas (FFA/PPA), while participants were unable to discriminate these categories, thereby satisfying the most rigorous criteria for establishing absence of awareness"

I don't quite understand how this conclusion is consistent with the general point of the paper as outlined in the abstract. To render stimuli objectively invisible, a stronger mask was used, and this stronger mask led to weaker stimulus-evoked activity patterns in all of the brain regions analyzed. But, even with objective invisibility, the authors still find above chance decoding in all of these regions, so doesn't this mean that even with stricter criteria and improved methods (objective instead of subjective invisibility), the extent of unconscious visual processing is quite vast? Perhaps I'm not understanding the authors' interpretation of (A) the existence of significant multi-voxel patterns vs. (B) magnitude differences between subj vs. obj invisibility. (A) seems more relevant to the general question of unconscious information processing while (B) seems more relevant to a methodological nuance. Indeed, on the following page, the authors state, "Notwithstanding these

limitations, our findings provide more conclusive fMRI evidence for unconscious processing of objectively invisible stimuli in human visual cortex."

My general concern here is that what is stated in the abstract doesn't really match up with this main conclusion. The abstract states, "These results demonstrate that the hypothesized extent of unconscious information processing is determined by the measurement approach. Furthermore, our data show that subjective and objective approaches are associated with different neural correlates of consciousness and thus have implications for neural theories of consciousness." But one of the main conclusions seems to be that decoding was still possible in a ton of areas (EVC, LOC, OPA, OFA, PPA, FFA), even for objectively invisible stimuli.

How does this finding match-up with the claims towards the end of the abstract? What are the implications for neural theories of consciousness, and how do these implications differ for obj-vis vs. obj-inv contrasts compared to subj-vis vs. subj-inv contrasts? The key question here is whether the neural results just change in magnitude or in kind when these different measurements of awareness are used.

**Response:** In our previous version of the manuscript, we focused mostly on the differences between objective and subjective invisibility, but we agree with the reviewer that evidence for unconscious processing was quite vast even for objectively invisible stimuli. In the revised manuscript, we have therefore updated the Abstract and the Discussion, so that one central conclusion is the existence of unconscious processing even for objectively invisible stimuli. In particular, both in the Abstract and in the first paragraph of the Discussion we now state, before discussing differences between objective and subjective invisibility, “We found significant category information even for objectively invisible stimuli in both early, lower-level visual areas and in higher-level visual cortex, although representations in anterior, category-selective ventrotemporal areas (FFA/PPA) were less robust.” One important difference between the two definitions of invisibility is that a posterior-anterior gradient, with greater category information in higher-level visual cortex, similar to visible stimuli, was evident only for subjectively invisible but not for objectively invisible stimuli. We now highlight this finding in the revised Abstract. According to the contrastive logic, in which the neural correlates of consciousness are revealed by contrasting visible with invisible stimuli, this posterior-anterior gradient (or amplification of category information in anterior areas) could be considered a hallmark of conscious representation only when adopting objective definitions, but not when adopting subjective definitions. In our view, this highlights how subjective and objective approaches result in different neural correlates of consciousness.

2. Why does figure 3b not include obj-vis vs. obj-inv? A major focus of this paper is about how objective versus subjective measures of awareness lead to different neural results. Why only show subj-vis vs. subj-inv and subj-inv vs. obj-inv? Obj-vis vs. obj-inv seems just as interesting of a contrast for the purposes of this study.

**Response:** Thank you for this suggestion. We have added the obj-vis vs. obj-inv contrast in Figure 3b (as well as in Figure S5b), and we are now referring to this additional contrast in the revised Results section (“Compared to this effect of subjective visibility, the contrast of objectively visible with objectively invisible stimuli revealed greater and more widespread differences in category information in overlapping occipito-temporal regions, as well as additional effects in inferior frontal regions.”).

3. On p. 8, first full paragraph, the authors state, "...participants did a simple one-back task on stimuli presented in a block design. This approach ... is often considered to isolate perceptual representations from later cognitive processes." I don't think this is the case. A one-back task requires subjects to hold each image in memory to see if it matches the next image. Holding things in memory seems like a "later cognitive process" and it's unclear how such a task isolates perceptual representations from memory processes.

**Response:** We agree – our statement was meant to refer to the correlation between the main experiment and the localizer rather than to the benchmark patterns from the localizer alone. As different tasks were adopted in these two parts of the study, multi-voxel pattern correlations cannot reflect cognitive processes specific to one of the two tasks. We changed the sentence to “This approach is statistically powerful and rules out the possibility that multi-voxel pattern correlations reflected cognitive processes related specifically to the task, decision, and motor processes (as these differed between the localizer and the main experiment).” We also changed a similar sentence in the Discussion from “Isolating perceptual representations in this way…” to “Ruling out task-specific representations in this way…”

4. Small but important typo on p. 5, second to last sentence in first paragraph of results: "(subj-vis: mean contrast 7.8%, SD 4.4; subj-vis: mean contrast 11.9%, SD 4.3)." I believe the first label should be "subj-inv".

**Response:** Thanks for pointing this out. Changed.

**Reviewer #3**

Major comments:

1. My first concern relates to the objective condition - typically, experimenters try to reach the sweet spot where the physical signal is as strong as possible, while objective performance is at chance. Although this was done here, to some extent, in the pilot experiment probing masking efficiency, it was not done well enough, I am afraid. The authors chose the maximal contrast of the mask based on the group performance in the pilot study Could it be that this manipulation is too strong, and substantially abolishes the signal evoked by the stimuli? A much preferable approach, which is often taken by researchers (e.g., Hesselmann et al., 2006, JoV) is to first calibrate the physical parameters (e.g., contrast) per subject, given the known differences between individuals, and find that sweet spot for each one. This is much more likely to allow higher-level processing also using the objective threshold. To take the current approach ad absurdum, one could also simply present the critical stimuli at 0% contrast, get objective chance performance and no signal whatsoever… This is of course not the case here (and indeed effects were found), but the authors should still explain why they chose not to calibrate per participant to allow for the strongest possible signal in the objective condition, and acknowledge that this could be the cause for the difference they find between the two conditions.

**Response:** Thanks for raising this interesting issue. In our revision (Results section, masking-efficiency experiment), we highlight our group-based approach for calibrating mask contrast and discuss individual calibration of mask contrast as an “alternative approach that is sometimes thought to be better suited to localize the “sweet spot” for unconscious processing”, allowing “for maximum signal strength for every participant”, citing the paper by Hesselmann et al., 2006, JoV. Our decision for group-based calibration was motivated by issues with establishing chance performance in previous studies using individual calibration and by the actual data from our masking-efficiency experiment. First, studies adopting individual calibration often fail to yield chance performance for all observers. This was also the case in Hesselmann et al., 2006, JoV, where several participants had to be excluded based on above-chance performance in the main experiment. Thus, individual calibration may underestimate awareness (for example, because a precise estimate of individual-subject thresholds may require thousands of trials). One of the main goals of our study was to avoid issues with post-hoc data selection in the study of unconscious processes, such as regression to the mean when participants are excluded post-hoc (Schmidt, 2015; Shanks, 2017). Second, the data from our masking-efficiency experiment revealed low between-subject variability: different observers performed in a narrow range for high mask contrast. At the same time, reliability for individual differences at high mask contrasts was low. This “reliability paradox” is well established for perceptual/cognitive tasks (Hedge et al., 2018). Low reliability implies that individual calibration at higher mask strengths would capitalize on noise. Our group-based approach, by contrast, benefitted from low between-subject variability. In the revised Supplement we report reliability estimates and between-subject variability in the masking-efficiency experiment, and describe our motivation for adopting group-based calibration (Figure S1b, Table S1). Although we consider group-based calibration superior for our sandwich masking procedure, individual calibration may be preferable for other presentation methods (such as continuous flash suppression). More generally, we cannot exclude the possibility that our group-based calibration missed the “sweet spot” for unconscious processing, and we now discuss this possibility in the Results section, also pasted below:

“Note that optimal mask contrast was determined based on the group data from the masking-efficiency experiment and set accordingly for the whole group of fMRI participants. An alternative approach that is sometimes thought to be better suited to localize the “sweet spot” for unconscious processing, is to calibrate mask contrast individually to allow for maximum signal strength for every participant50. However, the masking-efficiency experiment revealed low between-subject variability, as well as low reliability for the estimation of individual performance measures for high mask contrasts (see Supplementary Material). We thus considered group-based calibration of mask contrast better suited to ensure chance performance for all participants in the fMRI experiment, although we cannot exclude the possibility that our group-based calibration approach did not result in optimal stimulus strength for every individual observer.”

2. The above issue is even more problematic when taking into account the definition of subjective visibility, which acts in the opposite direction; if the objective threshold is too strict, the subjective one seems too lenient. The authors write that subjects were instructed to report the stimuli as visible "even when they had only a vague idea of the stimulus category", and accordingly, claim that "subjective invisibility on our binary scale corresponded to the lowest visibility level of more fine-grained visibility scales, such as the four-point Perceptual Awareness Scale". I am not convinced that this is accurate, as the second level in PAS is "A feeling that something has been shown. Not characterised by any content, and this cannot be specified any further" (Ramsøy Overgaard, 2004). Thus, having a vague idea of the stimulus category is actually more visible than the second level, where one should have *no* access to any content about the stimulus - they only know

something was there, but don't have any knowledge about what it was, not even a vague idea. This suggests that the "invisible" rating in this study actually reflects visibility levels 1 and 2, and not only 1. Unfortunately, this does pose a substantial problem, as the entire claim made here is based on the difference between subjective and objective threshold. But this comparison is now more problematic, as we are no longer comparing the lowest subjective invisibility with objective invisibility. I am not sure how this can be solved. To the very least, the authors should acknowledge this limitation and change the claims so to refer to the two lowest levels of subjective invisibility. The latter have been used in some studies to define invisibility (e.g., Melloni et al., 2011, J Neuroscience), but I wouldn't say it is the common practice in the field - typically, only the first level is used. Importantly, in many of these studies subjects are also at chance for the objective measures for visibility 1 trials - which is not the case here (and this might be explained by the fact that their "invisible" condition is not really invisible). Thus, I am not sure the critical test presented here - comparing the level of processing between objective and subjective invisibility - is a fair one.

**Response:** We thank the reviewer for highlighting these issues with the interpretation of subjective measures. We agree that it can be debated how our binary scale maps onto the four PAS categories. We therefore removed all claims regarding the mapping of the two scales from the manuscript (Results and Methods). Instead, we included a new paragraph in the Discussion section (also pasted below) where we follow the reviewer’s suggestion and acknowledge that “Depending on specific instructions and observer’s interpretations the present “invisible” rating may have corresponded to the lowest level or to the two lowest PAS levels. While some PAS studies defined invisibility as the two lowest PAS levels^75^, using only the lowest level is more common in the literature^20,21,23^. Compared to this approach, our subjective measure may have been more liberal in defining invisibility, resulting in greater estimates of blindsight-like above-chance performance for subjectively invisible stimuli.” In this paragraph, we also highlight that this illustrates a general problem with “private” subjective measures, which cannot be externally validated or falsified. Observer’s private interpretations and decision criteria may differ between specific scales, observers, experiments, and even trials. Given this context- and observer-dependency, it is simplistic to speak of “subjective (in)visibility.” As highlighted in our final paragraph of the Discussion “models where “unconscious processing” is seen as a unitary mechanism or concept, without further specifying how absence of awareness was established, will not provide a good fit to the full range of empirical data.” One solution to this problem is to adopt objective measures, which may come at the cost of sacrificing the subjective quality that is the core of consciousness, and which may reflect unconscious processes. Another potential solution for future studies that we highlight in the newly included paragraph is to adopt recently developed criterion-free measures of subjective awareness such as meta-d’ based on confidence ratings.

“Because it is the very nature of subjective measures that visibility judgements are subject to the observer’s own interpretation and response criterion, it is possible that results would have differed for the subjectively invisible condition, had we adopted another visibility scale. Although binary visibility scales such as the one used here have been widely adopted^26^, for example in studies using masking^70,71^, attentional blink^72^, dichoptic fusion^73^, and interocular suppression^74,75^, recently the four-point Perceptual Awareness Scale (PAS)^17^ has gained in popularity. Depending on specific instructions and observer’s interpretations the present “invisible” rating may have corresponded to the lowest level or to the two lowest PAS levels. While some PAS studies defined invisibility as the two lowest PAS levels^75^, using only the lowest level is more common in the literature^20,21,23^. Compared to this approach, our subjective measure may have been more liberal in defining invisibility, resulting in greater estimates of blindsight-like above-chance performance for subjectively invisible stimuli. This highlights a general problem with subjective measures. Subjective states are “private”, i.e. they cannot be externally validated or falsified, so that differences between scales, observers, or studies cannot be unequivocally linked to differences in perceptual states. One solution to this problem is to adopt objective measures, which may come at the cost of failing to capture the subjective quality of conscious experience and which may be influenced by unconscious processes. For these reasons, it has been notoriously difficult to determine whether above-chance performance for subjectively invisible stimuli reflects unconscious processing or a conservative response criterion in the subjective measure. One promising avenue for future studies is to adopt recently developed criterion-free measures of subjective awareness such as meta-*d’* based on confidence ratings^76^.”

3. I agree with the authors that this work is probably the most robust demonstration of unconscious processing in fMRI to date. Given the concerns I've raised above, I would emphasize this point more, and deemphasize the differences between the objective and the subjective conditions (as again, I think the comparison here is biased towards finding differences). The unconscious findings by themselves are highly interesting and important, and also set the bar for future studies aiming to make similar claims.

**Response:** We agree with the reviewer that the findings for the objectively invisible are interesting in themselves. Following the suggestions of both reviewers, in the revised manuscript we therefore updated the Abstract and the Discussion, so that one central conclusion is the existence of unconscious processing even for objectively invisible stimuli. In particular, both in the Abstract and in the first paragraph of the Discussion we now state, before discussing differences between objective and subjective invisibility, “We found significant category information even for objectively invisible stimuli in both early, lower-level visual areas and in higher-level visual cortex, although representations in anterior, category-selective ventrotemporal areas (FFA/PPA) were less robust.” For the comparison between objective and subjective invisibility, following the academic editor’s suggestions, we now focus on the difference in the posterior-anterior gradient between the two definitions of invisibility.

4. Was any correction for multiple comparisons across the paper? Such a correction is in order (e.g., FDR). The latter seemed to have been applied on the searchlight analysis, but there were many others conducted throughout the manuscript that should also be corrected for (for an explanation about the importance of correcting comparisons throughout the paper, see Benjamini et al., 2001, Behavioral Brain Research).

**Response:** We applied FDR correction to the whole brain searchlight maps. In these analyses, the same hypothesis is tested with many (thousands) of endpoints (voxels), making a correction necessary. However, the ROI analyses proceeded according to the standard approach of first running an omnibus ANOVA and subsequently following up with post-hoc tests in case of significant interaction effects. It is unclear how FDR (or any) correction should be adopted for these ANOVAs. The cited paper by Benjamini et al. (2001) specifically deals with studies that have many endpoints, such as in their example of behavioral genetics, where *N* groups are compared on many dependent variables (test batteries with approx. 100 endpoints), so that some effects turn out to be significant by chance (in this example, on average, 5). This situation would be similar to having 100 ROIs and testing all against chance. This is different from our ANOVA-based ROI analyses, where a set of post-hoc tests follows significant interaction effects. For the reviewer’s interest, if we took the (uncommon) approach of submitting all post-hoc p-values for a given ANOVA to an FDR correction, our conclusions would not change (p-values would just slightly increase). Rather than adopting this unconventional approach we have included Bayes factors for all statistical tests, as these do not suffer from potential issues with multiple testing.

5. When talking about objective measures potentially underestimating unconscious processing, the authors should also mention as one of the reasons that above-chance performance in the objective task might be driven by unconscious effects.

**Response:** Thanks for the suggestion. We included the sentence “Furthermore, above-chance performance in objective measures may be driven by unconscious processing” in the respective paragraph in the Discussion. We also mention this possibility in the newly included paragraph on subjective measures that we pasted above in response to point 2 (“One solution to this problem is to adopt objective measures, which may come at the cost of failing to capture the subjective quality of conscious experience and which may be influenced by unconscious processes.”)

6. The authors rightfully note that their sample size is larger than the typical fMRI studies in the field. But how was it determined? Was there any rationale behind it?

**Response:** The sample size was constrained by the resources provided by an fMRI grant awarded to the first author. We decided to test as many participants as possible within the allotted scanning time. We added this information in the Methods section.

Minor comments:

1. I find it somewhat strange that when talking about "all major theories of consciousness", IIT is not mentioned. If the authors think it does not assume unconscious processing (which I don't believe is the case), they should change the text to "most major theories of consciousness". Otherwise, I think it should be cited there as well.

**Response:** Thank you for highlighting our omission, our mistake. We added a reference to IIT (Tononi et al., 2016).

2. I apologize but I don't understand what is depicted in the line plots in figure 2. Are these simply lines connecting between the "peaks" of the bar plots? I don't really see why they are needed, as the readers can simply imagine them when looking at the bars (they are also somewhat misleading, as the variable is not continuous). I would remove them (or, if the authors really think they are necessary, add them to the bars plot. But I would advise to remove). If this is not what they depict, then please explain what is represented there.

**Response:** Thanks – we removed them.
