# Supplementary material for: The human visual system differentially represents subjectively and objectively invisible stimuli
Source: PLoS Biol. 2021 May 5;19(5):e3001241. doi: 10.1371/journal.pbio.3001241 (PMC8128378; doi:10.1371/journal.pbio.3001241)
Supplement: Attachment — Submitted filename: rebuttal2.docx [file pbio.3001241.s010.docx]

**Academic editor**

Editorially, we would like to make your title a bit more instructive, rather than descriptive, and suggest using: “The human visual system differentially represents subjectively and objectively invisible stimuli.” We would be happy to work with on a similar alternative.

**Response:** We changed the title to the suggested alternative.

Please indicate within your manuscript is your experiments were conducted according to the principles expressed in the Declaration of Helsinki or any other national or international ethical guideline.

**Response:** We added this information (Methods, participants section p. 16, 3rd paragraph: “The experiments were approved by the University of Amsterdam ethics committee, and all procedures were conducted according to the principles expressed in the Declaration of Helsinki.”).

Thank you for uploading your quantitative data to OSF. I could not find files for Fig S1, even when the figure legend indicates the data should be there. Could you please either explain or upload those data as well? Please disregard this point if the data were already uploaded but I missed them.

**Response:** Our omission, apologies. This has been fixed now – data underlying Figure S1 can be found on the OSF page: https://osf.io/qus5v/

**Reviewer #3**

1.      The running title is "subjective vs. objective measures of awareness", which in my opinion underrepresents the scope of the paper. I would change to: "processing subjectively vs. objectively invisible stimuli".

**Response:** We changed the running title accordingly.

2.      The addition of a disclaimer about the lack of individual calibration is excellent, but I wonder if readers who are not experts in the field would understand the key message. I accordingly suggest adding an explanation at the end. After "although we cannot exclude the possibility that our group-based calibration approach did not result in optimal stimulus strength for every individual observer", I would add: "This might have reduced the chances for finding higher-level processing also for objective-invisibility", or "This might have inflated the difference between objective invisibility and subjective invisibility in our study".

**Response:** We added both suggested statements at the end of that paragraph, as proposed by the reviewer (p. 6, 1st paragraph, last sentence: “This might have reduced the chances of finding higher-level processing also for objective invisibility, and might have inflated the difference between subjective and objective invisibility in our study.”).

3.      The figures were of low quality; I imagine that this is just some technical limitation of the review process, but write this warning to make sure this would not be the case for the published paper.

**Response:** We hope that this indeed a problem with the editorial web-based system, as we uploaded all figures in 300 dpi.
